# Inhibition of TGF-β signaling enables long-term proliferation of mouse primary epithelial stem/progenitor cells of the tympanic membrane and the middle ear mucosa

**DOI:** 10.1038/s41598-023-31246-y

**Published:** 2023-03-20

**Authors:** Tomomi Yamamoto-Fukuda, Filipa Pinto, Keshia Pitt, Makoto Senoo

**Affiliations:** 1 Department of Molecular and Cell Biology, Boston University Henry M. Goldman School of Dental Medicine, 72 East Concord Street, Boston, MA 02118 USA; 2 Department of Otorhinolaryngology, Jikei University School of Medicine, 3-25-8 Nishishinbashi, Minato-Ku, Tokyo 105–8461 Japan; 3 Cell Exosome Therapeutics, Inc., 2-16-9 Higashi, Shibuya-Ku, Tokyo 150-0011 Japan

**Keywords:** Cell biology, Stem cells

## Abstract

The surface of the middle ear is composed of the tympanic membrane (TM) and the middle ear mucosa (MEM). A number of diseases and conditions such as otitis media, middle ear cholesteatoma, and perforation of the TM have been reported to cause dysfunction of the middle ear, ultimately leading to high-frequency hearing loss. Despite its importance in repairing the damaged tissues, the stem/progenitor cells of the TM and the MEM epithelia remains largely uncharacterized due, in part, to the lack of an optimal methodology to expand and maintain stem/progenitor cells long-term. Here, we show that suppression of TGF-β signaling in a low Ca^2+^ condition enables long-term proliferation of p63-positive epithelial stem/progenitor cells of the TM and the MEM while avoiding their malignant transformation. Indeed, our data show that the expanded TM and MEM stem/progenitor cells respond to Ca^2+^ stimulation and differentiate into the mature epithelial cell lineages marked by cytokeratin (CK) 1/8/18 or Bpifa1, respectively. These results will allow us to expand epithelial stem/progenitor cells of the TM and MEM in quantity for large-scale analyses and will enhance the use of mouse models in developing stem cell-mediated therapeutic strategies for the treatment of middle ear diseases and conditions.

## Introduction

The middle ear is composed of multiple functional units, including the three auditory ossicles (incus, malleus, and stapes), the tympanic membrane (TM), the eustachian tube, and the middle ear cavity. The major function of the middle ear is to transfer the acoustic energy from the TM to the cochlea through the controlled middle ear-pressure. The dysfunction of the middle ear can lead to high frequency hearing loss^1^. Otitis media (ear infection) has been associated with hearing loss at the estimated global prevalence of approximately 30 (range 0.7–95) per 10,000 individuals with particularly high incidence in the developing countries^2^. When otitis media becomes chronic, perforation of the TM can occur which in turn results in varying degrees of perforation site-associated conductive hearing loss^3^.

The epithelial cells lining the surface of the middle ear, including TM epithelial cells (TMEC) and MEM epithelial cells (MEMEC), must self-renew to maintain tissue homeostasis and the barrier against bacterial infection, chemical damage, and mechanical injuries^4,5^. Abnormalities of epithelial stem/progenitor cells in the TM and the MEM can lead to the development of middle ear diseases such as otitis media, middle ear cholesteatoma, and perforation of the TM as has been suggested in previous studies in vitro and in vivo^6–8^. For example, we have shown that hyperproliferation of epithelial stem/progenitor cells of the TM associates with the pathogenesis of the middle ear cholesteatoma^7^. Other studies by us and Tucker et al*.* have shown that the dysfunction of epithelial stem/progenitor cells of the MEM causes otitis media with effusion^4,7^. Another study by Cho et al. has shown that blast-induced TM-perforation is associated with an increased risk of chronic hearing loss due to the dysregulation of homeostasis of the epithelial stem/progenitor cells in the TM^9^.

Mouse models have been used for studying stem cell function and developing stem cell-based therapeutic strategies in regenerative medicine. However, unlike the human cell counterpart^10^, the growth of mouse epithelial stem/progenitor cells rapidly declines in culture and the cells become terminally differentiated^11^, limiting the use of mouse primary epithelial stem/progenitor cells for functional studies. To circumvent this problem, we have recently developed a novel culture of mouse epithelial stem/progenitor cells^12^ by stabilizing p63, the transcription factor essential for maintaining the proliferative potential of stem cells in epithelia^13^, through the inhibition of the transforming growth factor-β (TGF-β) signaling that induces the terminal differentiation of epithelial stem/progenitor cells^14^.

Counteracting the function of the stem cell factor p63 in epithelia is mediated by the TGF-β signaling through the receptor complex consisting of the Type I TGF-β receptor (TGFβR1/ALK5) and the Type II TGF-β receptor (TGFβR2) that induce terminal differentiation of epithelial stem/progenitor cells^15^. It has been shown that TGF-β signaling pathway is operative in both MEM and TM^5,16,17^, suggesting that the coordinated balance between p63- and TGF-β signaling-pathways may control the self-renewal and differentiation of TM and MEM epithelia.

The purpose of this study was to determine whether the use of RepSox, a potent inhibitor of the TGF-β signaling (or alternative TGF-β inhibitors)^12^ could be used to propagate and maintain epithelial stem/progenitor cells of the TM and the MEM long term. As expression of p63 is the key to success in this novel epithelial stem/progenitor cell culture^12^, we first verified it in the prospective stem/progenitor cell areas of the TM and the MEM epithelia. Subsequently, we investigated whether the isolated TM and MEM epithelial stem/progenitor cells were capable of both self-renewal in Green’s co-culture with 3T3-J2 cells^10^ and differentiation into mature epithelial cell lineages by Ca^2+^ treatment^18,19^.

Our results show that the suppression of the TGFβ signaling in a low Ca^2+^ condition enables the long-term expansion and maintenance of p63-positive TM and MEM epithelial stem/progenitor cells while avoiding their malignant transformation. This methodology can produce a large quantity of p63-positive mouse primary TM and MEM epithelial stem/progenitor cells, allowing us to perform many downstream analyses at a large scale and at a clonal resolution long-term when combined with Green method. More importantly, these results will promote the use of mouse models to develop stem cell-based therapeutic options for middle ear diseases and conditions.

## Results

### Localization of p63-positive epithelial cells in the middle ear of adult mice

**Figure 1:**
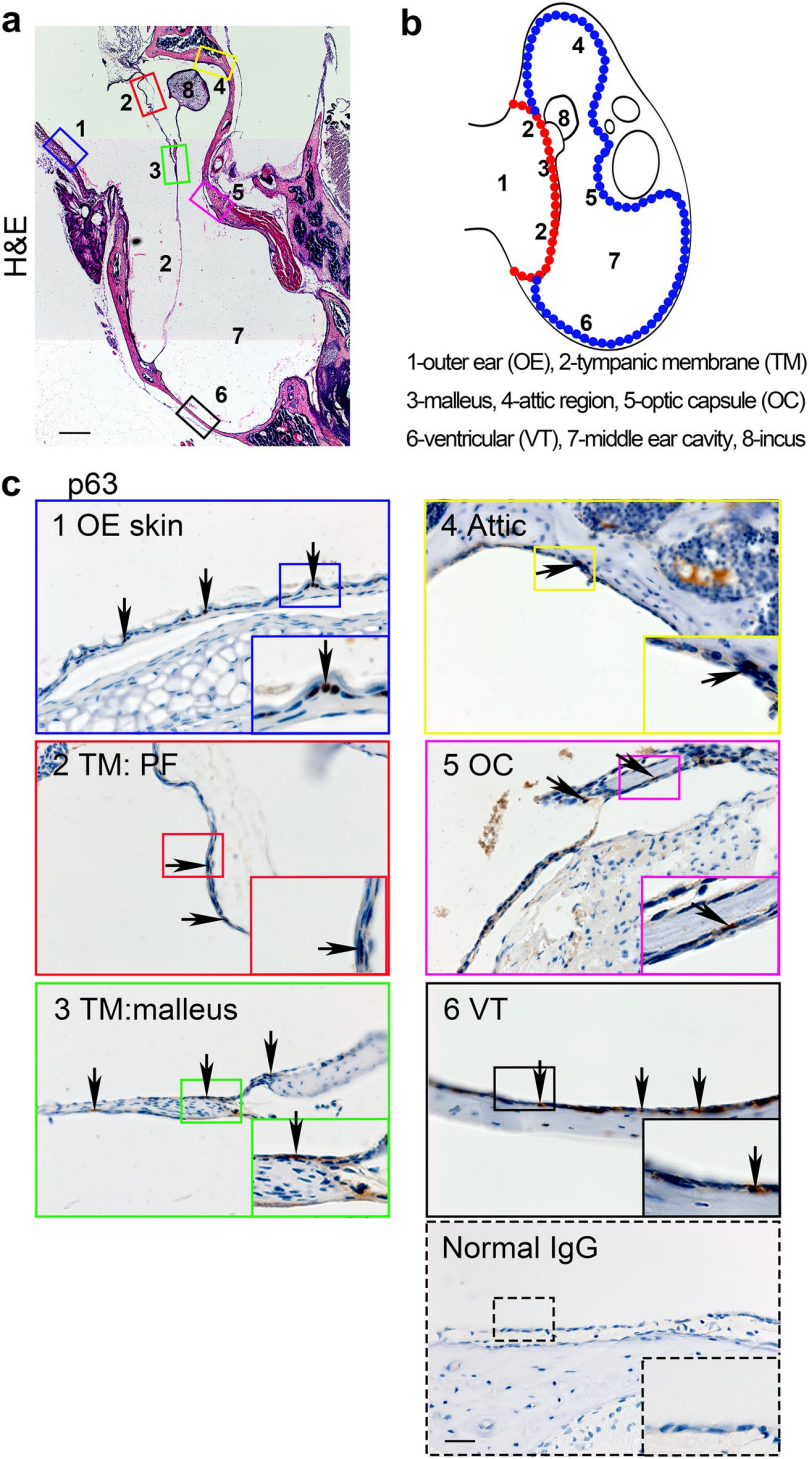
The localization of p63-positive epithelial cells in the mouse temporal bone. (**a**) A representative image of 5 week-old mouse temporal bone stained with hematoxylin and eosin (H&E). The boxes in blue, red, green, yellow, magenta, and black indicate the outer ear (OE), pars flaccida (PF) of the tympanic membrane (TM), malleus, the attic region, the optic capsule (OC), and the ventricular (VT) region, respectively. Scale bar = 1.0 mm. (**b**) A schematic illustration of the temporal bone in mice. The red dots indicate the epithelial layer of the TM, whereas blue dots indicate the epithelial lining of the middle ear cavity. (**c**) Immunostaining of 5 week-old mouse temporal bone stained with anti-p63 monoclonal antibodies. The arrows indicate representative p63-positive epithelial cells. Insets on the bottom right corner in each panel show higher magnification of the corresponding boxed area. The lower right panel shows an example of middle ear epithelium stained with normal rabbit IgG, serving as a negative control. Scale bar = 50 μm.

To determine the distribution of p63-positive epithelial cells in the middle ear, we performed immunohistochemical analysis of the sections of 5 week-old mouse temporal bones stained with anti-p63 antibodies. Figure 1a,b show a representative image of hematoxylin and eosin (H&E) staining of the middle ear and a schematic view of the corresponding structures, respectively.

The middle ear and the outer ear (OE) were separated by the TM (Fig. 1b, marked in red). The malleus, most lateral of the auditory ossicles, attached to the TM and articulated with the incus, the next auditory ossicle (Fig. 1a,b). The attic region, the optic capsule (OC), and the ventricular (VT) region were lined by the MEM (Fig. 1b, marked in blue). Analogous to the known localization of the stem cells in the skin^13^, p63-positive cells were found in the basal layer of the epidermis of the OE (Fig. 1c, blue boxes). Within the TM, p63-positive cells were found in the outer layer of the epithelium of the pars flaccida (PF) (Fig. 1c, red boxes) and an increased number of p63-positive cells were found in the epithelial layer near the malleus (Fig. 1c, green boxes). Some p63-positive cells were also found in the simple epithelium of the attic region near the TM (Fig. 1c, yellow boxes) and the OC region (Fig. 1c, magenta boxes). The p63-positive cells in the MEM in the VT region were predominantly found in the basal layer of the epithelium (Fig. 1c, black boxes). No staining was detected in the control slides processed with the class-matching normal IgG (Fig. 1c, dotted box as an example). As it has been shown that mice deficient in p63 display severely impaired formation of the middle ear structures^20^, our results suggest that the identified p63-positive cells likely play a key role in the development of the TM and the MEM epithelia.

### Inhibition of the TGF-β signaling in a low Ca^2+^ condition enables the expansion of mouse primary TM and MEM epithelial stem/progenitor cells in vitro

We have shown previously that suppression of TGF-β signaling in a low Ca^2+^ condition enables the long-term proliferation of p63-positive epithelial stem/progenitor cells of many different tissues and organs in mice^12,21^. The involvement of the TGF-β signaling in this principle was obvious as not only RepSox but also many other TGF-β signaling inhibitors showed similar effect on epithelial stem/progenitor cell expansion^12^. Expression of high levels of p63 is prerequisite for the expansion in our novel culture, as its growth condition itself does not induce de novo expression of p63 in p63-negative cells or rejuvenated differentiating epithelial cells with reduced p63 levels^12^.

**Figure 2:**
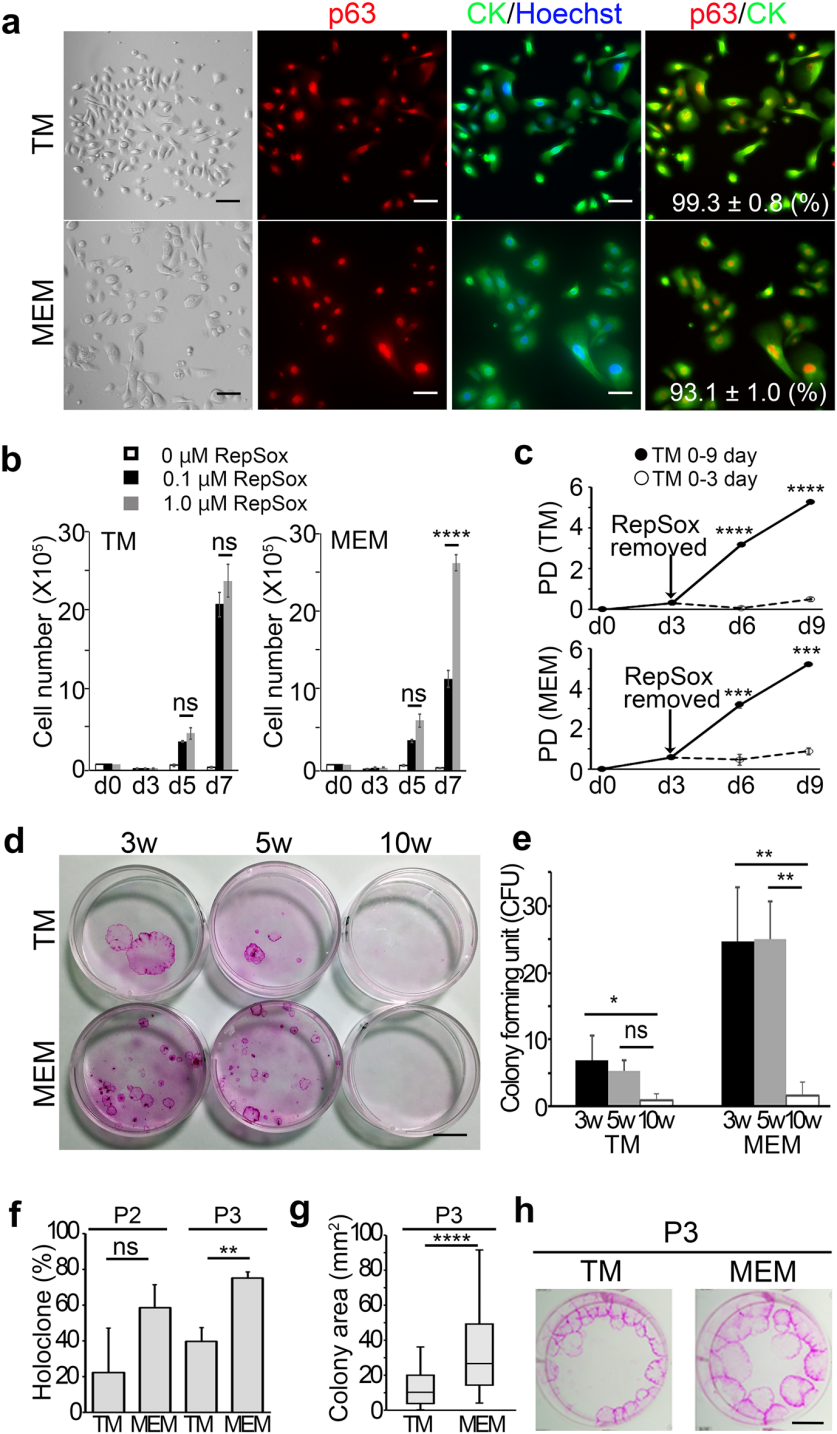
Inhibition of TGF-β signaling enables long-term proliferation of p63-positive stem/progenitor cells of the TM and the MEM epithelia in vitro. (**a**) Representative brightfield images captured on a phase-contrast microscope (far left). Fluorescence images on the right show representative immunofluorescence staining with anti-p63 antibody alone (2nd panels), anti-pan-cytokeratin (CK) antibody counterstained with Hoechst 33,342 (3rd panels), and anti-p63 antibody and anti-pan-CK antibody (far right). Immunofluorescence images were captured with a widefield epifluorescence scope (BZ-X710) with a Nikon Plan Fluor 20×/0.50 objective lens. Top and bottom rows represent data from the TM and the MEM culture, respectively. The primary cells were grown in the presence of 0.1 μM RepSox. Scale bars = 20 μm. The labeling index in the right panels was expressed as the percentage of double positive cells for p63 and pan-CK expression at passage 2 (n = 4). (**b**) Growth of the stem/progenitor cells of the TMEC and MEMEC in CnT-PR media for 7 days in the absence (open) or presence of 0.1 µM (closed) or 1.0 µM (hatched) RepSox. The cells were used at passage 5 (P5). Data shown are mean ± S.D. (n = 4). ****P < 0.0001. *ns*, non-significant. (c) Population doubling (PD) of RepSox-expanded epithelial stem/progenitor cells of the TM and the MEM grown in CnT-PR media in the presence of RepSox (TM: 0.1 μM, MEM: 1.0 μM). The cells were used at passage 7 (P7). The dotted lines indicate the reduced cellular growth when RepSox was removed at day 3 (d3) of culture. Data shown are representative of three independent experiments with similar results. ****P < 0.0001, ***P < 0.001. (**d**) Rhodamine B staining of 3, 5, and 10 week-old mouse-derived primary TMECs and MEMECs (P0) grown in Green’s co-culture with 3T3-J2 cells in the presence of 0.1 μM RepSox for 14 days. Data shown are representative of three independent experiments with similar results. Scale bar = 10 mm. (**e**) Colonies forming unit (CFU) in co-culture shown in (**d**). Closed columns; 3 week-old mouse, hatched columns; 5 week-old mouse, open columns; 10 week-old mouse. Data shown are expressed as mean ± S.D. (n = 4). **P < 0.01, *P < 0.05. *ns*, non-significant. (**f**) Percentage of holoclones derived from the TM and the MEM in co-culture with 3T3-J2 at P2 and P3 cultivated in the presence of 0.1 μM RepSox. (n = 3). **P < 0.01. *ns*, non-significant. (**g**) Box Plot showing the distribution of the size of TM- and MEM-derived epithelial cell colonies in co-culture with 3T3-J2 cells in the presence of 0.1 μM RepSox (TM: n = 89, MEM: n = 64). ****P < 0.0001. (**h**) Representative images of Rhodamine B staining of the clonogenic culture of the TM and the MEM epithelia with 3T3-J2 cells at P3 grown in the presence of 0.1 μM RepSox. Data shown are representative of three independent experiments with similar results. Scale bar = 10 mm.

Given that both TM and MEM harbor p63-positive cells (Fig. 1), we postulated that suppression of TGF-β signaling in a low Ca^2+^ condition would also allow some of these epithelial stem/progenitor cells of the TM and the MEM to expand. To address this possibility, we prepared crude explants of the TM and the MEM from 5 week-old mice and grew them in CnT-PR, a chemically-defined low Ca^2+^ medium, in the presence of RepSox (Supplementary Fig. S1). The cells with typical epithelial morphology outgrew the explants rapidly, and virtually all the cells became cytokeratin (CK)-positive and p63-positive (CK^+^p63^+^) by passage 2 (P2) (99.3 ± 0.8% in the TM culture and 93.1 ± 1.0% in the MEM culture) (Fig. 2a).

RepSox treatment robustly enhanced proliferation of the CK^+^p63^+^cells of the TM and the MEM (Fig. 2b). Our data show that CK^+^p63^+^ cells of the TM are somewhat more sensitive to the RepSox treatment than those of the MEM at a lower dose of 0.1 µM (Fig. 2b). Our data also show that the expansion of CK^+^p63^+^cells of both TM and MEM was RepSox-dependent as removal of RepSox completely abolished the proliferation of both cell types (Fig. 2c). We also tested other widely used TGF-β signaling inhibitors and found that RepSox was the most effective on both TM and MEM epithelial stem/progenitor cell proliferation (Supplementary Fig. S2). This is consistent with our previous study with other epithelial types^12^, such as the skin, cornea, oral epithelia, salivary glands, thymus, esophagus, trachea, and bladder, showing that RepSox has the highest activity among all the TGF-β signaling inhibitors across all the epithelial cell types we have tested.

In our previous study, we showed that the frequencies of clonogenic stem/progenitor cell populations as determined by Green’s co-culture with 3T3-J2 cells^10^ decline with age in all epithelial tissues and organs tested^12^. Consistent with these findings, the frequencies of clonogenic stem/progenitor cells of the TM and the MEM declined sharply in 10 week-old mice compared to 3 and 5 week-old mice (Fig. 2d,e). These data showing the rapid decrease of the stem cell fraction of the TM and MEM are in good agreement with previous findings by Anthwal and Thompson, showing that the middle ear development in mice starts during embryogenesis and the maturation of the TM and the MEM epithelium continues to occur until the third week after birth^22^, while the number of stem/progenitor cells of the TM and the MEM decreased dramatically by 8th week after the birth^23^.

To determine the self-renewal capability of the RepSox-expanded epithelial stem/progenitor cells of the TM and the MEM, we employed Green’s co-culture with 3T3-J2 cells, and performed a serial cultivation at a clonal level with two-week intervals in the presence or absence of RepSox. Our results show that while in the absence of RepSox epithelial cells no longer formed macroscopically visible clones in the subsequent passage (data not shown), the presence of RepSox continuously produced holoclone-like colonies at similar rates for at least three generations in both TM and MEM cultures (Fig. 2f,h). Our data also show that the MEM culture produced relatively larger clones than TM culture (Fig. 2g,h). The difference in clone sizes might be attributable to cell-intrinsic differences in stem/progenitor cells, their differential responses to the paracrine signaling mediated by 3T3-J2 feeder cells, or a combination of both^24^.

These results indicate that continuous suppression of the TGF-β signaling in serial 3T3-J2 co-culture allows us to assess self-renewal of mouse TM and MEM epithelial stem/progenitor cells.

### The ability of differentiation of the TM and the MEM epithelial stem/progenitor cells expanded by the RepSox protocol

To determine whether TM and MEM epithelial stem/progenitor cells expanded by the RepSox protocol are capable of differentiation, we performed Ca^2+^-mediated differentiation assays in vitro^12,18^. To induce differentiation, we stimulated the cells with CnT-PR media without RepSox and supplemented with an elevated level of Ca^2+^ to 1.3 mM. Mouse skin epidermal stem/progenitor cells were used as a positive control to monitor morphological changes during differentiation.

**Figure 3:**
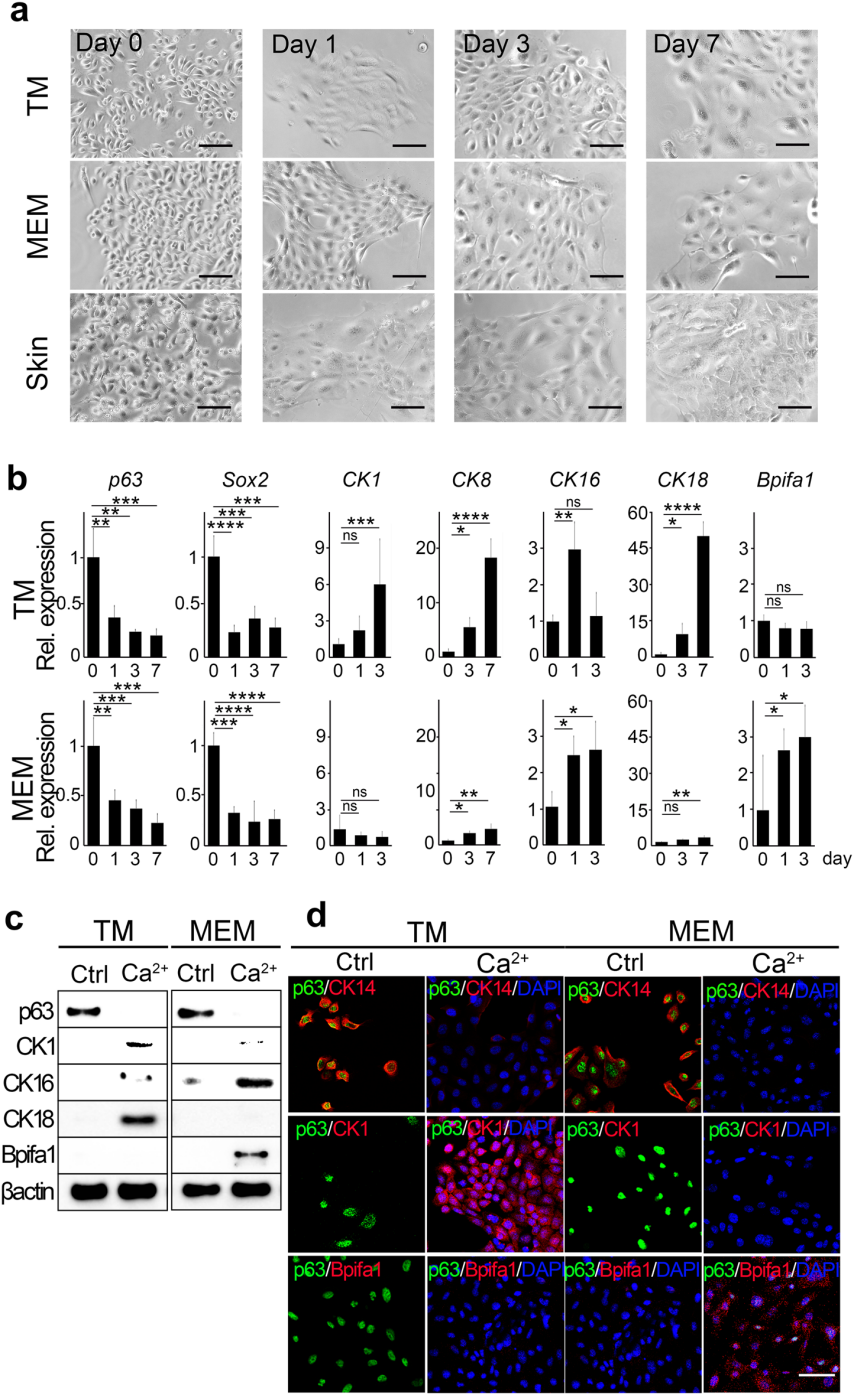
The expanded mouse primary epithelial stem/progenitor cells of the TM and the MEM are capable of differentiation in response to Ca^2+^ stimulation in vitro. (**a**) Representative images of 3 week-old mouse-derived, RepSox-expanded primary epithelial stem/progenitor cells of the TM and the MEM before (Day 0) and after stimulation with 1.3 mM Ca^2+^ for 1, 3, and 7 days as indicated. Newborn mouse-derived, RepSox-expanded P18 skin epidermal cells (lower panels) were used as a control to assess morphological changes of epithelial cells during Ca^2+^-mediated differentiation. Scale bars = 100 μm. (**b**) Quantitative RT-PCR analysis of epithelial cell differentiation marker genes. RepSox-expanded, mouse primary epithelial stem/progenitor cells of the TM and the MEM were induced to differentiate by the treatment with 1.3 mM Ca^2+^ for 0–7 days as indicated. Data shown are normalized to the housekeeping gene *Gapdh* and expressed as mean ± S.D. (n = 3). *P < 0.05, **P < 0.01, ***P < 0.001, ****P < 0.0001. *ns*, not significant. (**c**) Western blot analysis using antibodies against p63, CK1, CK16, CK18 and Bpifa1. RepSox-expanded, mouse primary epithelial stem/progenitor cells of the TM and the MEM were induced to differentiate by the treatment with 1.3 mM Ca^2+^ for 0 (Ctrl) and 3 (Ca^2+^) days. β-actin was used as a loading control. (d) Double-immunofluorescence detection of p63 (green) and CK14 (red, undifferentiated epithelial cell marker), CK1 (red, differentiated epithelial cell marker) or Bpifa1 (red, ciliated mucosal epithelial cell marker) in RepSox-expanded, mouse primary epithelial stem/progenitor cells of the TM and the MEM before (Ctrl) and after the treatment with 1.3 mM Ca^2+^ for 3 days (Ca^2+^). No staining was observed when the primary antibodies were replaced with the species- and class-matching normal IgG (see Supplementary Fig. S4). Nuclei were counterstained with DAPI as indicated (blue). Scale bar = 20 μm.

Our data show that, similar to the skin epidermal stem/progenitor cells, the TM and the MEM stem/progenitor cells changed their morphology from a round sphere-like appearance to the cobblestone-like pattern typical for epithelial cells as early as day 1 post-differentiation (Fig. 3a). The changes in morphology were accompanied by those in expression of epithelial cell differentiation marker genes (Fig. 3b). For example, expression of *p63* and *Sox2*, two representative, well-characterized stem cell-related genes^12,25^, decreased upon differentiation in both TMEC and MEMEC (Fig. 3b). Decrease in p63 protein levels was verified by Western blot using whole cell extracts of TMEC and MEMEC at day 3 post-differentiation (Fig. 3c). Immunofluorescence staining shows that expression of p63 and of CK14, another commonly used epithelial stem/progenitor cell marker^26^, decreased to undetectable levels upon differentiation in virtually all the cells in both TMEC and MEMEC cultures (Fig. 3d). Subsequently, we analyzed the expression of epithelial cell differentiation markers including those that are unique to each TM and MEM lineage. Transcription of *CK1*, an early and late differentiation marker of epithelial cells^27,28^, increased in TMEC but not in MEMEC in response to Ca^2+^ stimulation (Fig. 3b). Likewise, transcription of *CK8* and *CK18*, differentiated simple epithelial cell markers^29^, also increased in TMEC in response to Ca^2+^ stimulation (Fig. 3b). In contrast, transcription of *CK16*, a hyperproliferative epithelial cell marker^30,31^ increased in both TMEC and MEMEC although the upregulation was transient in TMEC (Fig. 3b). Transcription of *Bpifa1*, a ciliated mucosal epithelial cell marker^32,33^, increased in MEMEC but not in TMEC in response to the stimulation with Ca^2+^ (Fig. 3b). The changes in gene expression of *CK1, CK16, CK18,* and *Bpifa1* were reflected at the protein level as determined by Western blot (Fig. 3c and Supplementary Fig. S3) and immunofluorescence analysis (Fig. 3d and Supplementary Fig. S4).

**Figure 4:**
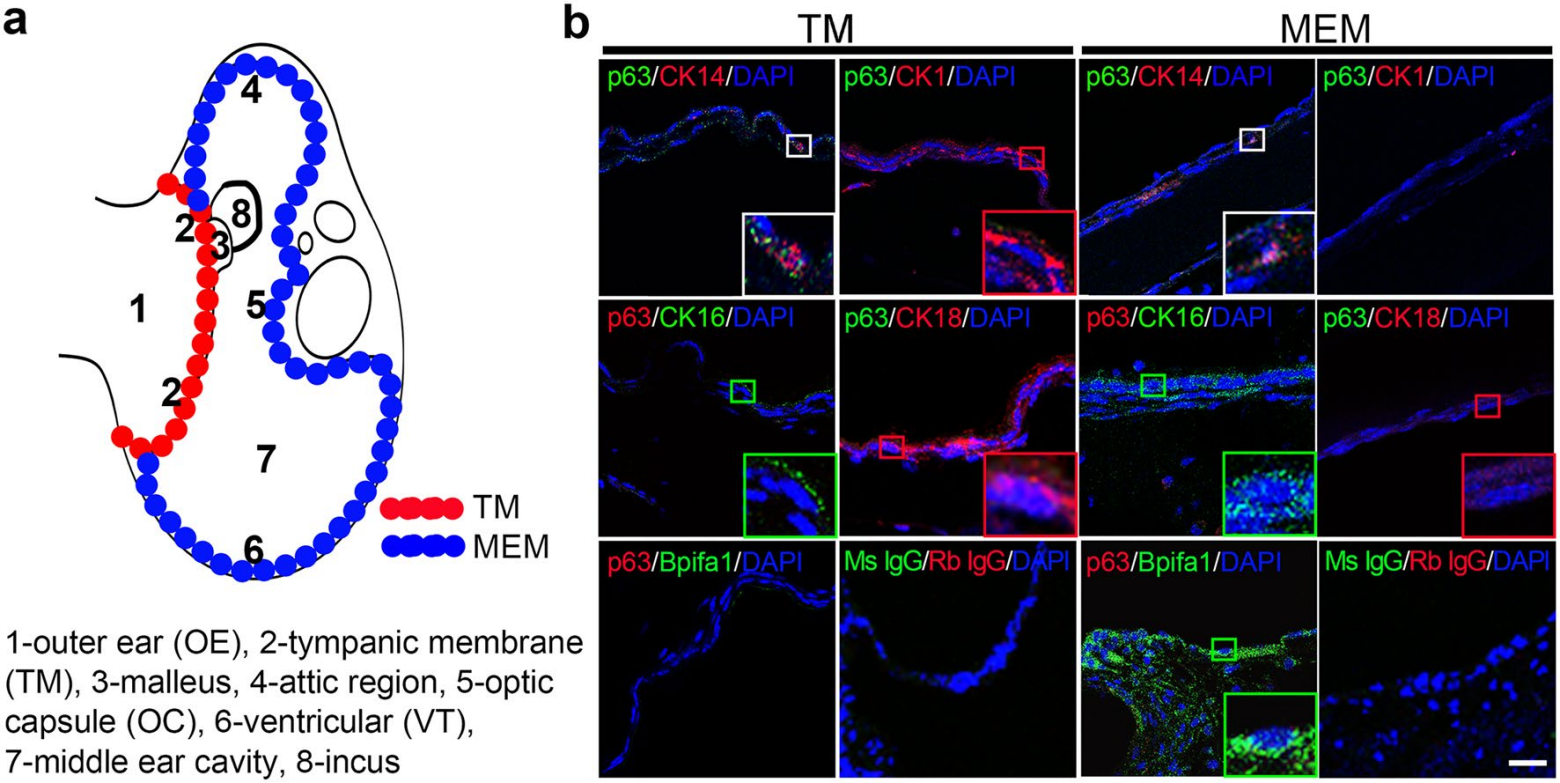
Expression of p63 and other epithelial cell differentiation markers in vivo. (**a**) A schematic illustration of the mouse temporal bone. The red and blue dashed lines indicate the TM and the MEM, respectively. (**b**) Immunofluorescence staining of p63, CK14, CK1, CK16, CK18 and Bpifa1 in paraffine sections of 5 week-old mouse ear tissues. Insets on the bottom right corner show a higher magnification of the boxed area in the same panel. Fluorescence images were captured using fluorescence confocal laser scanning microscopy LSM 880 with a Plan-Apochromat 40×/0.95 Korr M27 NA objective lens. Scale bar = 20 µm. No staining was observed when normal mouse IgG or rabbit IgG was used as the primary antibodies. Nuclei were counterstained with DAPI (blue).

Finally, we verified physiological expression of CK1, CK14, CK16, CK18, and Bpifa1 in relation to p63 expression in the TM and the MEM of adult mice (Fig. 4a,b). Consistent with its function as stem/progenitor cells in epithelia, a small number of double-positive cells for p63 and CK14 was detected in the basal layer of the TM and the MEM epithelia (Fig. 4b). We found that the cells positive for CK1, CK16, CK18, and Bpifa1 in the TM and the MEM epithelia were all negative for p63 as they mark differentiated epithelial cell layers. For example, CK1-positive cells localized to the outer surface of the TM while CK18-positive cells were found more broadly in the TM epithelia except the basal layer (Fig. 4b). CK16-positive cells were detected in the suprabasal layer of the VT region of the MEM while Bpifa1-positive cells were detected in the attic region and the VT region near the Eustachian tube (Fig. 4b). These results indicate that expressions of the markers used in the Ca^2+^-mediated differentiation correlate well with those in the TM and the MEM epithelia in vivo.

Collectively, these results indicate that mouse TM and MEM epithelial stem/progenitor cells expanded by the RepSox protocol are capable of differentiation in response to Ca^2+^ stimulation. In addition, the differentiation markers analyzed in this study can be utilized as lineage markers for distinguishing TM and MEM epithelial stem/progenitor cells upon differentiation.

## Discussion

In this report, we have shown that suppression of the TGF-β signaling in a low Ca^2+^condition enables long-term expansion of p63-positive mouse primary stem/progenitor cells of the TM and the MEM epithelia. To the best of our knowledge, this is the first report of long-term culture and maintenance of mouse primary TM and MEM epithelial stem/progenitor cells. Our approach is simple and cost effective as it does not require labor intensive stem/progenitor cell-enrichment steps (e.g. via fluorescence-activating cell sorting) or support by a feeder cell layer as we have reported for the other epithelial stem/progenitor cell types^12^.

The transcription factor p63 plays an essential role in controlling the proliferative activity of epithelial stem/progenitor cells during development, homeostasis, and regenerative processes in response to wounding^13,34^. In the previous studies, stem/progenitor cells of the TM were identified in the outer epithelial layer of the manubrium, annulus and handle of the malleus by lineage tracing and assessment of the growth factor-mediated proliferative response *in vivo*^35,36^. When the TM was punctured in 4 to 5 week-old mice, proliferative cells were predominantly detected around the annulus and handle of the malleus adjacent to the TM, indicating that the TM stem/progenitor cells localize in these specific domains of the TM^36^. In the present study, we show that p63-positive cells were enriched in epithelial cell layers of the TM nearby the malleus (Fig. 1), suggesting that the cellular environment near the malleus may provide a niche for self-renewing stem/progenitor cells of the TM. In support, a significant epithelial cell outgrowth was detected from the minimally excised explants of the TM that surrounded the malleus (Supplementary Fig. S1). Another group has described the stem cells of the TM as those that are positive for integrin β1, a common epithelial stem cell marker^37^. It is interesting to determine whether assessment with double staining of p63 and integrin β1 can be used for determining the hierarchy of the stem cells in the TM.

In our previous studies, we have described the MEM stem/progenitor cells as CK5/14-positive cells in the basal layer of the MEM as they played a role in maintaining MEMEC^34^. Tucker et al*.* have shown that stem/progenitor cells of the MEM in mice were enriched in the eustachian tube up until 2 weeks after birth and that such stem/progenitor cells of the MEM became rarely detectable at a later stage of adulthood beyond 3 weeks after birth and into adulthood^4^. In contrast, we have detected p63-positive cells in the basal layer of the MEM in the attic region and the VT region with close proximity to the eustachian tube in 5 week-old mice (Fig. 1). These results indicate that stem/progenitor cells of the MEM can be detected at a later stage of adulthood beyond the 2-week timepoint using anti-p63 antibodies. Indeed, tissue harvest of the MEM from 5 week-old mice produced a significant number of proliferative epithelial clones in Green’s clonogenic culture (Fig. 2d,e).

Stem cells proliferate in response to the inflammatory signaling in the damaged tissues. We have shown previously that p63-positive stem/progenitor cells were induced to proliferate in the attic, OC, and VT regions of the MEM in response to the administration of keratinocyte growth factor (KGF), a well-characterized inflammatory cytokine, in 6 to 8 week-old adult mice^38^. These data suggest that the specific regions of the MEM, such as the attic, OC, and VT regions, harbor stem/progenitor cells of the MEM. Our current results support this notion as the explant of the attic and VT regions of the MEM near the annulus and eustachian tube of the 5 week-old mice produced significant epithelial cell outgrowth (Supplementary Fig. S1).

Our data show that RepSox was superior to the other commonly used TGF-β signaling inhibitors (TGFβR1/ALK5 kinase inhibitors) in expanding p63-positive stem/progenitor cells of the TM and the MEM epithelia (Supplementary Fig. S2). However, the moderate inhibitory activity of RepSox against TGFβR1/ALK5 kinase in cell-free assays does not explain the superiority of RepSox (the IC_50_ of RepSox, LY364947, and SB525334 are 23 nM, 59 nM, and 14.3 nM, respectively). It would be possible that the unique biological/biochemical property of RepSox as the “replacement of the stem cell factor Sox2” (hence, the name RepSox)^39^ may play a role in expanding p63-positive stem/progenitor cells of the TM and the MEM epithelia. In this regard, it is important to note that Sox2 has been reported to stimulate the *p63* gene expression in corneal epithelial stem/progenitor cells^40^, suggesting that RepSox treatment may enhance the gene programs regulating epithelial stem cell self-renewal through the p63-dependent pathways.

Our data show that while the proliferative response of the TM culture reached plateau at a relatively low concentration of RepSox treatment (0.1 µM), the MEM culture required tenfold higher dose of RepSox (1.0 µM) to achieve the same cellular output in monolayer cultures (Fig. 2b,c). These results suggest that the basal level of the Sox2-mediated p63-dependent self-renewal activity of stem/progenitor cells is lower in the MEMEC than TMEC. Interestingly, Xie et al. have shown recently that the concentration of RepSox used for expanding neural stem cells was significantly lower than what was used for intestinal epithelial cells or fibroblasts^41^. Neural stem cells express high Sox2 levels^42^, and we predict that RepSox-mediated activation of the Sox2 signaling pathway can be used to promote the expansion of not only p63-positive epithelial stem/progenitor cells but also other unrelated Sox2-positive stem/progenitor cell types.

In contrast to the monolayer culture (Fig. 2b,c), epithelial stem/progenitor cells of the MEM in Green’s co-culture with 3T3-J2 feeder cells showed more rapid expansion than those of the TM in response to 0.1 µM RepSox treatment as determined by the colony forming unit, holoclone frequency, and colony size (Fig. 2d-h). These seemingly contradictory results to those in monolayer culture (Fig. 2b,c) may originate from the feedback loop by the feeder cells in co-culture. It has been shown that epithelial stem/progenitor cells produce Wnt ligands and they play an essential role in promoting epithelial stem cell self-renewal in an autocrine manner^43^. However, as we have shown previously, epithelia-derived Wnt ligands have a paracrine effect on the feeder cells and convert their growth-supportive state with insulin-like growth factor 2 (IGF2) production into the growth-inhibitory state accompanied by the induced production of TGF-β^24^. It would be important to determine the expression of IGF receptors and TGF-β receptors in the epithelial stem/progenitor cells of the TM and the MEM in future studies. Such determination will help not only for improving the current methodology of expanding the stem/progenitor cells of the TM and the MEM epithelia but also for developing therapeutic strategies of terminating abnormal stem/progenitor cell proliferation in diseases or activating quiescent stem cells to proliferate for promoting tissue regeneration.

Owing to their unique properties (e.g. self-renewal, proliferation and differentiation long-term), stem cells are being used in more than five hundred clinical trials in the United States alone^44^. However, the stem cell therapy of the middle ear still has a logistical barrier. For example, Sagiv et al. performed explant culture of human TM tissues and showed that both epithelial cells and fibroblasts could be outgrown but only for a limited period of time^45^. Liew et al. have established primary cells of rat TM tissues by explant cultures^46^. However, their primary cells were a mixed population of fibroblasts and epidermal cells^46^. Yamamoto et al. have established autologous nasal mucosal epithelial sheets suitable for transplantation into the middle ear mucosa^47^. They isolated nasal mucosal epithelial cells using trypsinization and cultured them on temperature-sensitive culture inserts. In this study autologous nasal mucosal epithelial cells differentiated into cytokeratin (CK)-positive stratified epithelial cells but not into the ciliated mucosal cells in the middle ear^47^.

Our novel approach has brought several technical advantages toward the long-term goal of therapeutic regeneration of the middle ear epithelia. First, it is a simple procedure. We can obtain highly enriched (> 99% purity) population of stem/progenitor cells of the TM and the MEM epithelia. Second, this methodology will provide p63-positive epithelial stem/progenitor cells in quantity. Third, the expanded stem/progenitor cells of the TM are capable of differentiation into CK1-positive stratified epithelial cells and CK18-positive mucosal epithelial cells, while those of the MEM differentiate into Bpifa1-positive ciliated mucosal epithelial cells^32,33^. However, it is critical to determine if the same principle reported here can be used to expand human TM and MEM stem/progenitor cells for therapeutic purposes.

In conclusion, we have shown that inhibition of TGF-β signaling using the small molecule inhibitor RepSox in a low Ca^2+^ condition selectively expands p63-positive mouse primary stem/progenitor cells of the TM and the MEM epithelia in quantity with the capability of differentiation. It is tempting to speculate that our results will enhance the use of mouse models for developing stem cell-mediated therapeutic strategies for middle ear diseases and conditions, and that the expanded p63-positive epithelial stem/progenitor cells can be used for establishing in vitro models of middle ear diseases.

## Materials and methods

### Animals

All experimental procedures were carried out in accordance with the guidelines by the Institutional Animal Care and Use Committees (IACUC) at the Boston University, and all experimental protocols in this study were approved by the Boston University IACUC (Protocol number: PROTO201800401, PI: Senoo). The reporting in this manuscript followed the recommendations in the ARRIVE guidelines (PLoS Bio 8(6), e1000412, 2010). Mice used in this study were on a C57BL/6 background (Jackson Laboratories, Bar Harbor, ME; Charles River Laboratories, Wilmington, MA). We followed the American Veterinary Medical Association (AVMA) Guidelines for the Euthanasia of Animals (2020).

### Tissue isolation and cell culture

Wildtype C57BL/6 mice were euthanized by isoflurane inhalation, followed by the decapitation. Mouse primary epidermal cells were prepared and seeded onto a non-coated dish containing CnT-PR basal media (Cellntec, Bern, Switzerland) supplemented with 1.0 μM TGF-β inhibitor (RepSox) (Cayman Chemical, Ann Arbor, MI) as described previously^12^. Primary cells of mouse TM and MEM were prepared by primary explant culture, as described previously^38,48–50^ with some modifications. The tissues harvested were sterilized in 100 U/mL penicillin and 100 µg/mL streptomycin (Gibco Invitrogen, Carlsbad, CA) in phosphate buffered saline (PBS). The temporal bones were dissected and the TM and the cup-shaped ME-bullae were dissected under a dissecting microscope (Nikon, Melville, NY). The ME-bullae attached to mucosa were minced into small pieces with surgical scissors. The TM and minced ME-bullae were seeded onto a non-coated dish in CnT-PR media supplemented with 0.1 μM RepSox. By passage 3, virtually all growing cells in culture were pan-CK-positive epithelial cells as determined by immunohistochemistry. The cells were grown in the presence or absence of 0–1.0 μM TGF-β inhibitors (RepSox, LY364947 and SB525334) (all from Cayman Chemical) as indicated. All primary cell cultures were performed in a humidified chamber at 37 °C with 5% CO_2_.

To determine the cell numbers, the growing cells in the plates were incubated in 0.25% trypsin for 8–10 min, neutralized with 10% fetal bovine serum (FBS) in DMEM and washed in PBS, followed by cell counting in a glass hemocytometer chamber (Hausser Scientific, Horsham, PA) under a CKX41 Inverted Microscope (Olympus, Center Valley, PA) with a 10 × objective. To measure population doubling, the cells were seeded at a density of 4 × 10^4^ cells per well in 12-well plates, grown in CnT-PR media in the presence or absence of 0.1–1.0 μM RepSox, and culture media were replaced every 24 to 48 h (hrs), followed by cell harvest at the time points indicated.

To induce differentiation in epithelial stem/progenitor cells of the TM and MEM, sub-confluent cells in 12-well plates were washed twice in PBS and cultivated in CnT-PR media containing 1.3 mM CaCl_2_ for 1–7 days as indicated. In the differentiation assays, culture media were replaced every 24 to 48 h and the cells were harvested at the time points indicated for subsequent analyses.

### 3T3-J2 co-culture

Clonogenic culture with 3T3-J2 cells was performed as described^11^. Briefly, mouse epithelial cells were seeded at a clonal density on mitomycin C (MMC, 10 µg/ml)-treated 3T3-J2 cells (gift from H. Green, Harvard Medical School) in complete FAD (cFAD) media and grown in the absence or presence of 0.1 μM RepSox in a humidified chamber at 37 °C with 5% CO_2_. To perform serial passages, epithelial cells were harvested from the initial 3T3-J2 co-cultures by trypsinization in 0.25% trypsin for 5–10 min and equal numbers of epithelial cells were serially cultured with MMC-treated fresh 3T3-J2 cells with two-week intervals. To visualize epithelial colonies, culture plates were fixed in 10% buffered formalin and stained with 1% Rhodamine B (Sigma-Aldrich, St. Louis, MO). To determine clone sizes, the whole plates were photographed with a Nikon digital camera Coolpix S10 (Nikon) and the diameter of each clone was measured with the aid of Adobe Photoshop (Adobe, San Jose, CA) software.

### Immunohistochemistry and Immunofluorescence

Immunofluorescence (IF) and immunohistochemistry (IHC) were performed as described^7,51^. Briefly, cultured cells were fixed in 10% buffered formalin and permeabilized in 0.1% Triton X-100 in PBS, followed by blocking with 10% FBS and incubation with primary antibodies. After three washes in 0.1% Tween-20 in PBS, the cells were incubated with either fluorescence-labeled secondary antibodies followed by counterstaining with 0.1 μg/ml Hoechst 33,342 (Invitrogen, Carlsbad, CA) or DAPI (Sigma-Aldrich, St. Louis, MO), or horseradish peroxidase (HRP)-conjugated secondary antibodies followed by detection of the antigens using a diaminobenzidine (DAB) reagent kit (KPL, Gaithersburg, MD).

The paraffin sections (5 µm thick) of temporal bones were prepared as we reported previously^52^ and some representative slides were stained with hematoxylin and eosin for histological evaluation^53^. Enzymatic- and fluorescence-based IHC was performed as described previously^7,54,55^. Antigen retrieval was performed by autoclaving the slides in HistoVT One at 90 °C for 20 min. The endogenous peroxidase activity was inactivated by treating the slides with 0.3% H_2_O_2_ in methanol. To block nonspecific binding, the slides were preincubated with 500 µg/ml normal goat IgG in 1% bovine serum albumin (BSA) in PBS. The slides were then incubated with the primary antibodies followed by a washing step and then incubation with appropriate secondary antibodies. The color reaction was developed with a DAB kit (KPL). For IF staining, the nuclei were counterstained with DAPI. For a negative control, normal mouse IgG (1:100) or normal rabbit IgG (1:100) was used as the primary antibodies. Detailed information about the antibodies used in this study are summarized in Supplementary Table S1.

### Western blotting

The cells (4.0 × 10^5^ cells) were lysed in 2 × Laemmli buffer (Bio-Rad, Hercules, CA) supplemented with 2-mercaptoethanol, a protein/phosphatase inhibitor cocktail (Cell Signaling Technology, Danvers, MA) as described previously^56^. Equal amounts of proteins were separated by 4–12% polyacrylamide gel and transferred to polyvinylidene difluoride membranes (Invitrogen). The membranes were blocked with 5% skim milk in 0.1% Tween 20 (in tris buffered saline), rinsed, and incubated with the primary antibodies. The antibody-antigen complexes were detected by incubating with the secondary antibodies. The signals were visualized using the ECL reagent (GE Healthcare Biosciences, Pittsburg, PA) and the images were captured using LAS 4000 Chemiluminescence Imager (GE Healthcare). Quantification was performed using the NIH ImageJ software ver. 15.1 (https://imagej.nih.gov/ij/)^57^ and the relative signal intensity was determined by normalizing with β-actin signal. Antibody information is summarized in Supplementary Table S1.

### RNA isolation and quantitative RT-PCR

Total RNA was isolated using Trizol reagent according to the manufacturer’s instructions (NucleoSpin® RNA, TaKaRa Bio, Shiga, Japan) and 1 μg total RNA was reverse-transcribed using the PrimeScript™ II 1st strand cDNA Synthesis Kit (TaKaRa Bio, Shiga, Japan). Quantitative PCR was performed using the SYBR Green PCR Master Mix on a Rotor-Gene Q/RG-6000 (Qiagen, Hilden, Germany) according to the manufacturer’s instructions (Qiagen). Relative expression of each gene was determined using the ΔΔCt method and normalized to the housekeeping gene *Gapdh*. Primer sequences used in this study are summarized in Supplementary Table S2.

### Image analysis

Images of stem/progenitor cells (Fig. 2) were captured using a Nikon Eclipse 80i microscope with a Nikon DS-Qi1Mc digital camera (Nikon) or a BZ-X710 microscope (Keyence Corp., Osaka, Japan) with a Nikon Plan Fluor 20x/0.50 lens (Nikon). To determine the p63-positive and the CK-positive cell numbers, randomly selected areas were photographed (n = 3 to 5 fields per sample) and the captured immunofluorescence images were processed with Adobe Photoshop software. Counterstaining with Hoechst 33,342 was used to determine the total cell numbers. Images of the H&E staining and enzymatic immunostaining (Fig. 1) were captured using an Axio Cam camera and AxioVision software version 4.8 (Carl Zeiss, Jena, Germany) under light microscopy. Fluorescence images in Figs. 3 and 4 were captured using fluorescence confocal laser scanning microscopy LSM 880 with a Plan-Apochromat 20x/0.8 M27 NA objective for differentiation assays and Plan-Apochromat 40x/0.95 Korr M27 NA objective for tissue sections, followed by analysis using Zen Black software (Carl Zeiss, Jena, Germany).

### Statistical analysis

Means and SDs were calculated from numeric data. Differences between the groups were examined for statistical significance using one-way analysis of the variance test, followed by the analysis of unpaired t-test or Tukey post hoc test for normally distributed data, where *P* < 0.05 was considered statistically significant. All analyses were performed using the JMP statistical software package version 13 (SAS Institute Japan, Tokyo, Japan).

## Supplementary Information

Supplementary Information 1.

Supplementary Information 2.

Supplementary Information 3.

Supplementary Information 4.

Supplementary Information 5.

## Data Availability

All data generated or analyzed in this study are included in this published article and its Supplementary Information files.
