# Supplementary figures and images for: Inhibition of TGF-β signaling enables long-term proliferation of mouse primary epithelial stem/progenitor cells of the tympanic membrane and the middle ear mucosa
Source: Sci Rep. 2023 Mar 20;13:4532. doi: 10.1038/s41598-023-31246-y (PMC10027825; doi:10.1038/s41598-023-31246-y)

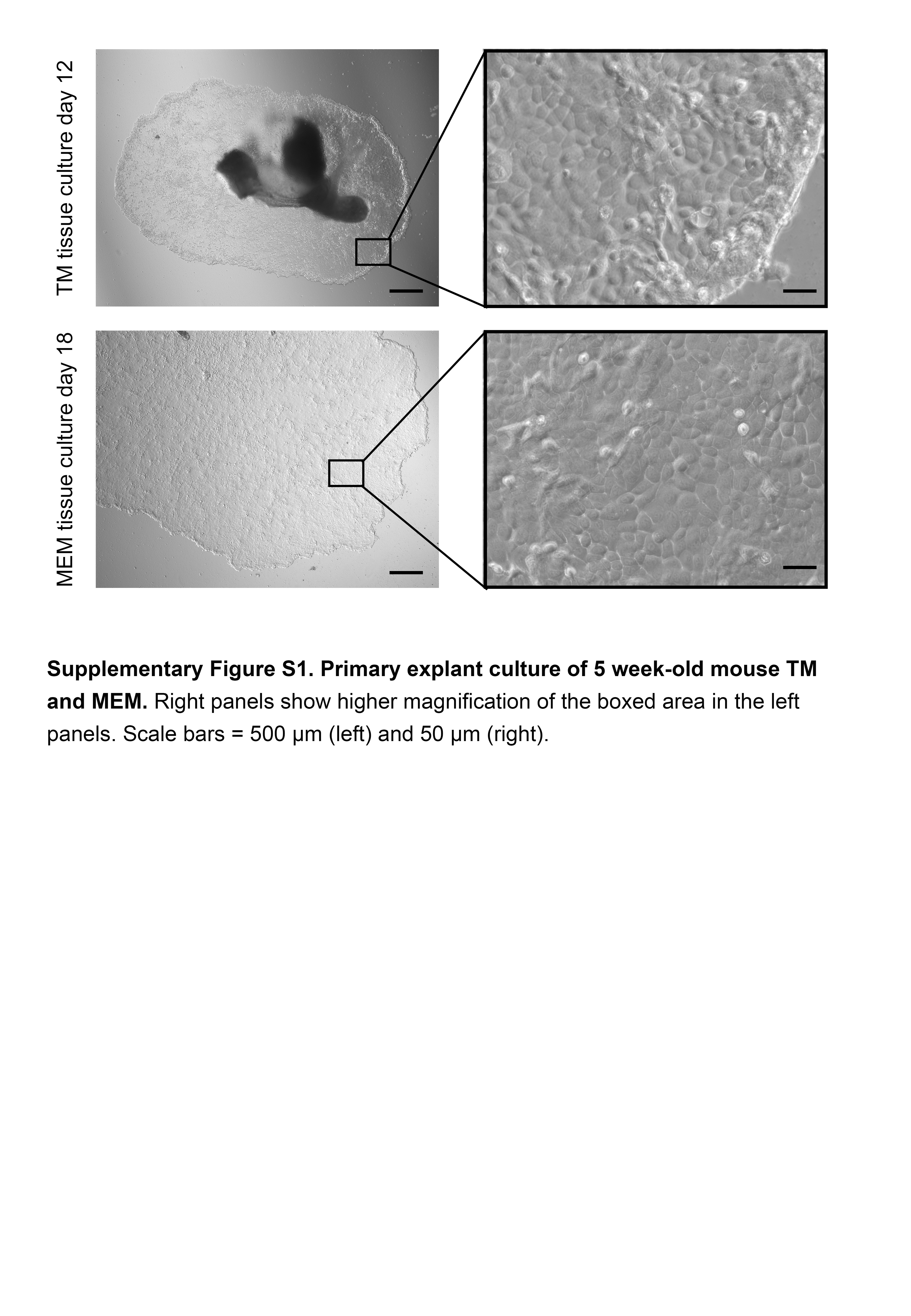

Supplement: Supplementary file 1 — Supplementary Information 1. [file 41598_2023_31246_MOESM1_ESM.jpg]

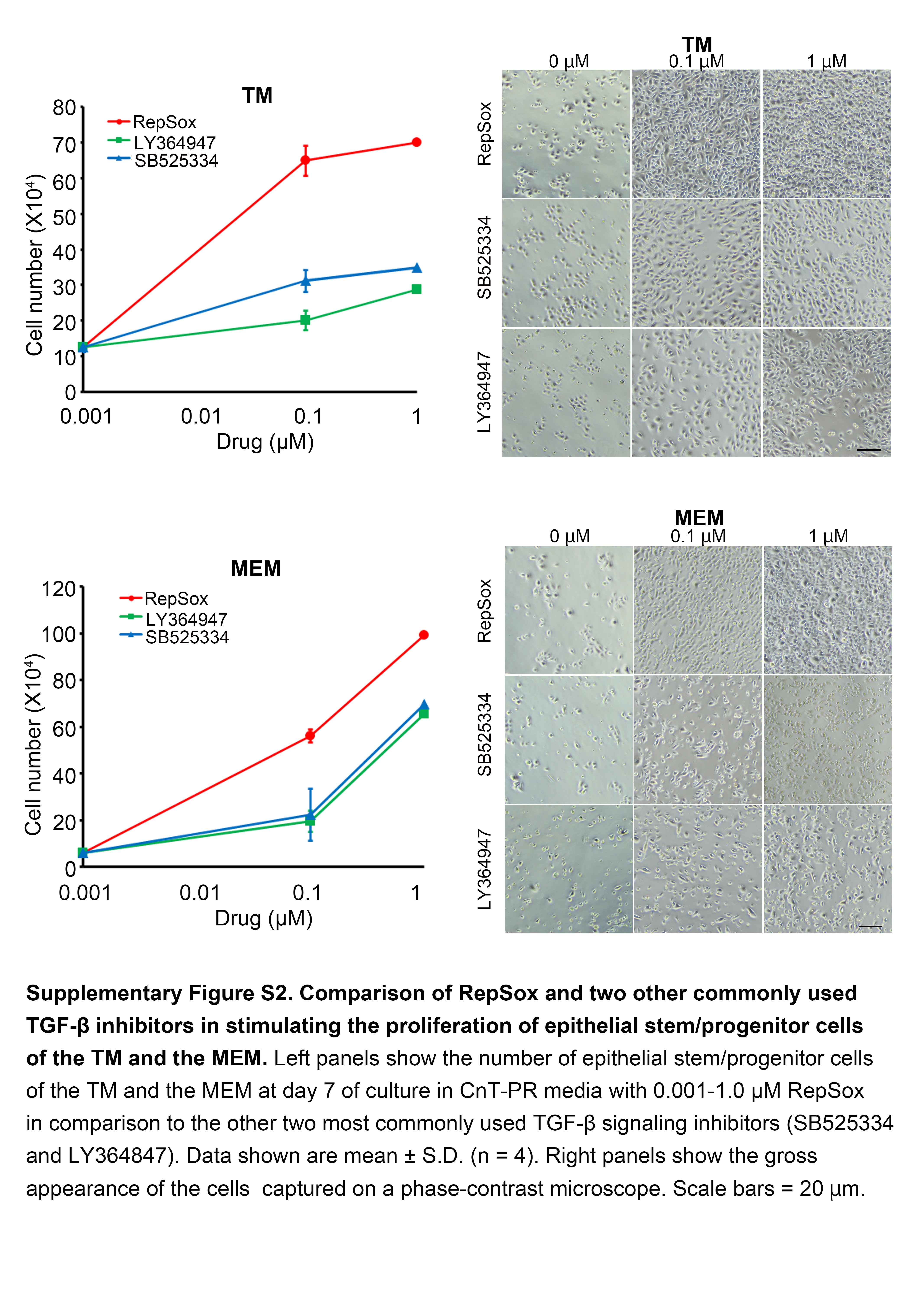

Supplement: Supplementary file 2 — Supplementary Information 2. [file 41598_2023_31246_MOESM2_ESM.jpg]

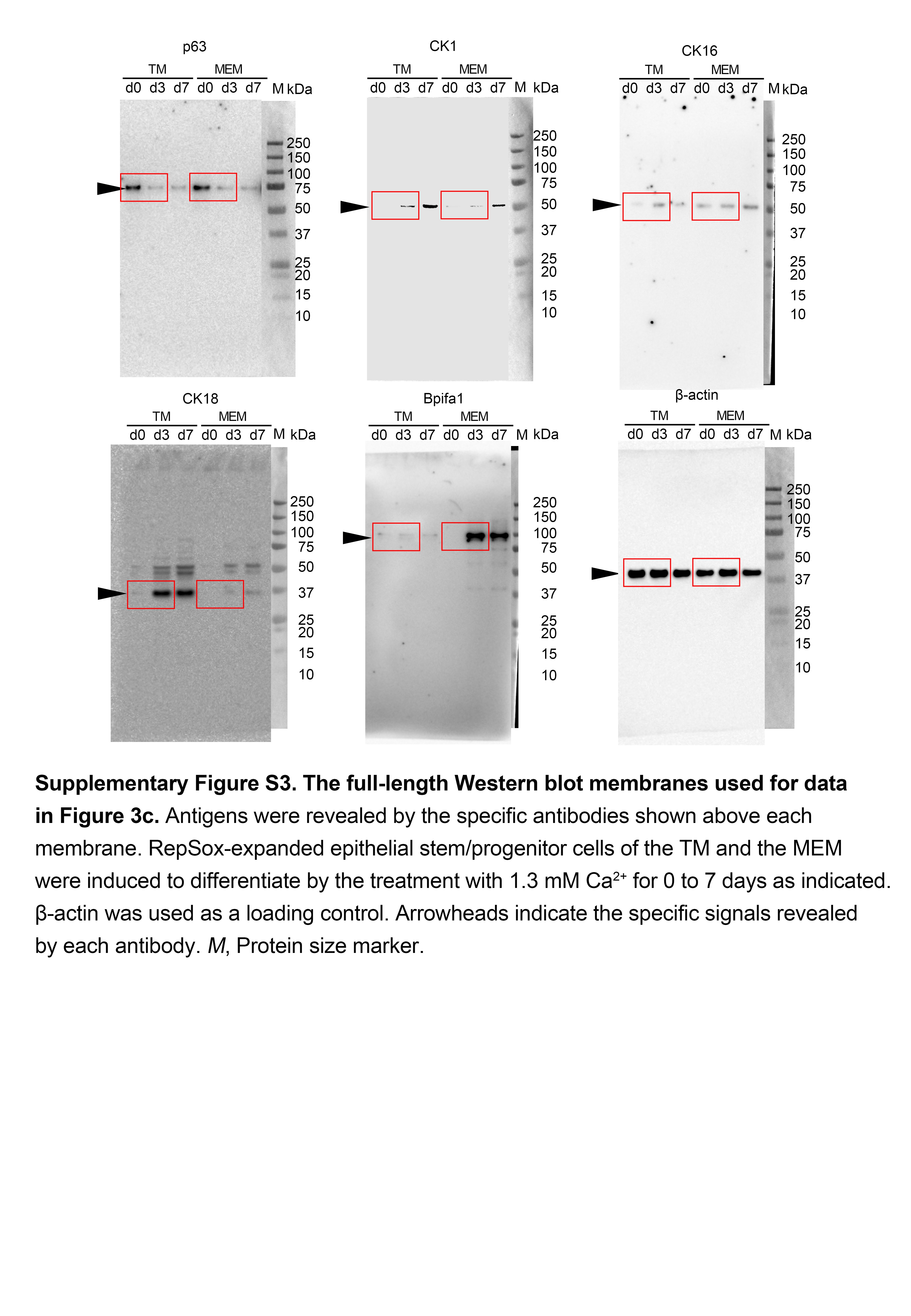

Supplement: Supplementary file 3 — Supplementary Information 3. [file 41598_2023_31246_MOESM3_ESM.jpg]

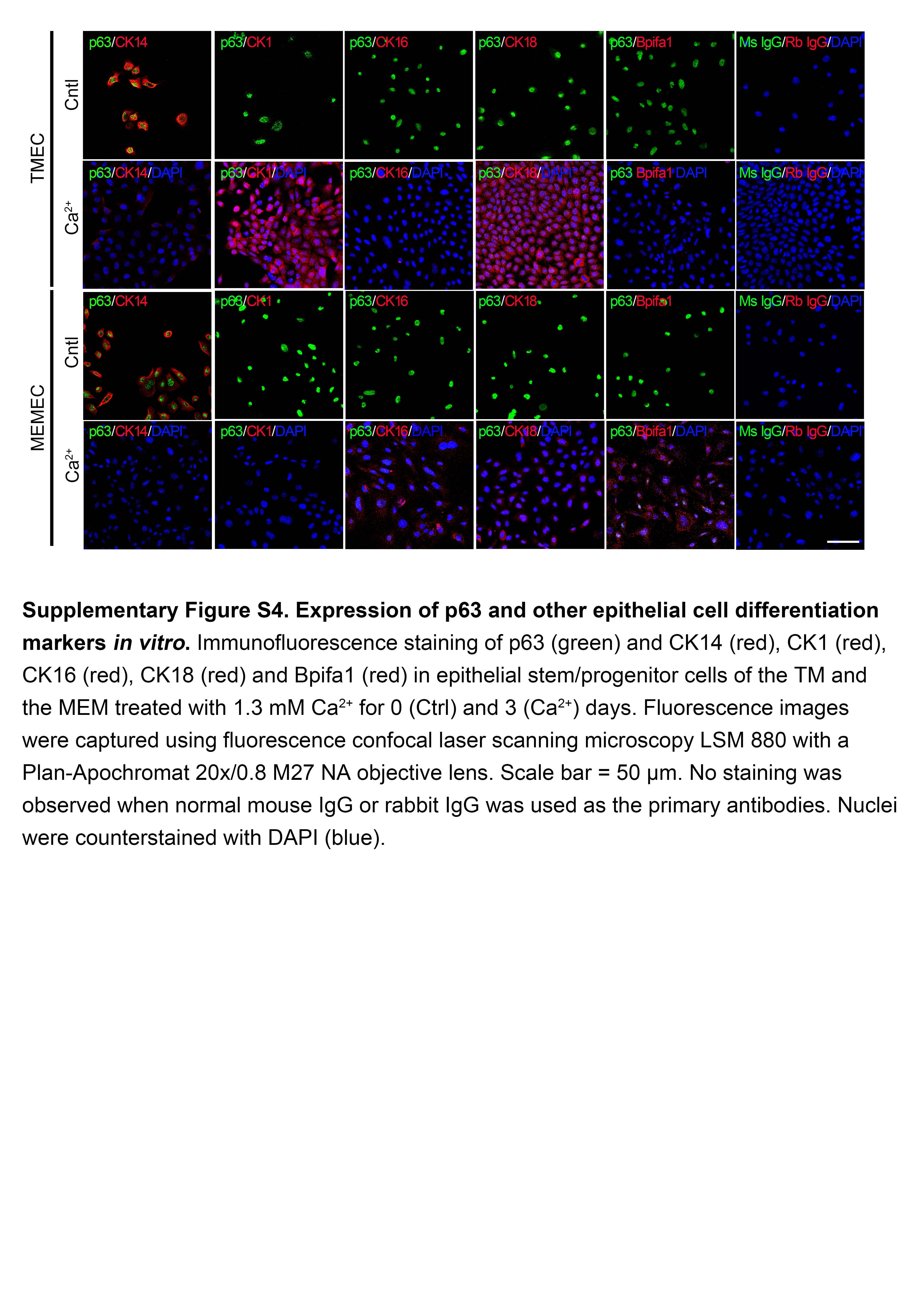

Supplement: Supplementary file 4 — Supplementary Information 4. [file 41598_2023_31246_MOESM4_ESM.jpg]
