# Supplementary material for: Inhibition of TGF-β signaling enables long-term proliferation of mouse primary epithelial stem/progenitor cells of the tympanic membrane and the middle ear mucosa
Source: Sci Rep. 2023 Mar 20;13:4532. doi: 10.1038/s41598-023-31246-y (PMC10027825; doi:10.1038/s41598-023-31246-y)
Supplement: Supplementary file 5 — Supplementary Information 5. [file 41598_2023_31246_MOESM5_ESM.docx]

Supplementary Table S1. Antibodies Used for Immunohistochemistry (IHC), Immunofluorescence (IF), and Western blotting (WB).

| Antigen, clone name or immunogen | Manufacturer, species, catalog no. | Dilution used | Type |
| --- | --- | --- | --- |
| p63, 4A4 | Santa Cruz Biotechnology, Santa Cruz, CA, mouse, sc-8431 | 1:1,000 IF/WB | Primary |
| p63, EPR5701  pan-CK, AE1/AE3 | Abcam, Cambridge, UK, rabbit, ab124762  Thermo Fisher Scientific, Waltham, MA, mouse, MS-343 | 1:200 IF; 1:2,500 IHC  1:100 IF | Primary  Primary |
| CK1, Polyclonal | BioLegend, San Diego, CA, rabbit, PRB-165P | 1:2,000 IF; 1:1,000 WB | Primary |
| CK8, M20  CK14, AF64  CK16, LL025 | Santa Cruz Biotechnology, mouse, sc-52324,  Covance, Berkeley, CA, rabbit, PRB-155P  Thermo Fisher Scientific, mouse, MA5-13730 | 1:50 IF; 1:400 WB  1:2,000 IF  1 µg/ml IF; 2 µg/ml WB | Primary  Primary  Primary |
| CK18, Polyclonal | Biosis, Boston, MA, rabbit, bs-2043R | 1:100 IF; 1:300 WB | Primary |
| SOX2, Polyclonal | Abcam, rabbit, ab97959 | 1:500 IF | Primary |
| BPIFA1, PLUNC [G-7] | Santa Cruz Biotechnology, sc-398364 | 1:50 IF; 1:400 WB | Primary |
| Beta-actin,13E5 | Cell Signaling Technology, Danvers, MA, rabbit, #4970 | 1:1,000 WB | Primary |
| Alexa Fluor 488-goat anti-mouse IgG | Thermo Fisher Scientific, goat, A-11001 | 1:1,000 IF | Secondary |
| Alexa Fluor 488-goat anti-rabbit IgG | Thermo Fisher Scientific, goat, A-11034 | 1:1,000 IF | Secondary |
| Alexa Fluor 594-goat anti-rabbit IgG | Thermo Fisher Scientific, goat, A-11037 | 1:1,000 IF | Secondary |
| HRP-goat anti-rabbit IgG | Abcam, goat, ab6721 | 1:250 IHC | Secondary |
| HRP-goat anti-mouse IgG | Cell Signaling Technology, mouse, #7076 | 1:10,000 WB | Secondary |
| HRP-goat anti-rabbit IgG | Cell Signaling Technology, rabbit, #7074 | 1:10,000 WB | Secondary |

*CK*, cytokeratin ; *HRP*, horseradish peroxidase

Supplementary Table S2. Primer sequences used for quantitative RT-PCR in this study.

| gene | Forward primer sequences (5’ to 3’) | Reverse primer sequences (5’ to 3’) |
| --- | --- | --- |
| *p63* | TGCCCAGACTCAATTTAGTG | TGGAGCTGGGCTGTGCATAG |
| *Sox2* | TAGAGCTAGACTCCGGGCGATGA | TTGCCTTAAACAAGACCACGAAA |
| *CK1* | TGGGAGATTTTCAGGAGGAGG | GCCACACTCTTGGAGATGCTC |
| *CK8* | TCCATCAGGGTGACTCAGAAA | CCAGCTTCAAGGGGCTCAA |
| *CK16* | ATGCACAGTTCACTTTGCAGA | ATGCACAGTTCACTTTGCAGA |
| *CK18* | ACTCCGCAAGGTGGTAGATGA | TCCACTTCCACAGTCAATCCA |
| *Bpifa1* | ACAGAGGAGCCGACGTCTAA | CCAAGAAAGCTGAAGGTTC |
| *Gapdh* | AACTTTGGCATTGTGGAAGG | CACATTGGGGGTAGGAACAC |
